# Immunological effects of the PE/PPE family proteins of *Mycobacterium tuberculosis* and related vaccines

**DOI:** 10.3389/fimmu.2023.1255920

**Published:** 2023-09-27

**Authors:** Fangzheng Guo, Jing Wei, Yamin Song, Baiqing Li, Zhongqing Qian, Xiaojing Wang, Hongtao Wang, Tao Xu

**Affiliations:** ^1^ Research Center of Laboratory, Bengbu Medical College, Bengbu, China; ^2^ Anhui Province Key Laboratory of Immunology in Chronic Diseases , Bengbu Medical College, Bengbu, China; ^3^ Department of Immunology, School of Laboratory, Bengbu Medical College, Bengbu, China; ^4^ Anhui Province Key Laboratory of Clinical and Preclinical Research in Respiratory Disease, Bengbu Medical College, Bengbu, China; ^5^ Department of Clinical Laboratory, School of Laboratory, Bengbu Medical College, Bengbu, China

**Keywords:** *Mycobacterium tubercolosis*, PE/PPE family, vaccine, tuberculosis, immunology

## Abstract

Tuberculosis (TB) is a chronic infectious disease caused by *Mycobacterium tuberculosis* (*Mtb*), and its incidence and mortality are increasing. The BCG vaccine was developed in the early 20th century. As the most widely administered vaccine in the world, approximately 100 million newborns are vaccinated with BCG every year, which has saved tens of millions of lives. However, due to differences in region and race, the average protective rate of BCG in preventing tuberculosis in children is still not high in some areas. Moreover, because the immune memory induced by BCG will weaken with the increase of age, it is slightly inferior in preventing adult tuberculosis, and BCG revaccination cannot reduce the incidence of tuberculosis again. Research on the mechanism of *Mtb* and the development of new vaccines against TB are the main strategies for preventing and treating TB. In recent years, Pro-Glu motif-containing (PE) and Pro-Pro-Glu motif-containing (PPE) family proteins have been found to have an increasingly important role in the pathogenesis and chronic protracted infection observed in TB. The development and clinical trials of vaccines based on *Mtb* antigens are in progress. Herein, we review the immunological effects of PE/PPE proteins and the development of common PE/PPE vaccines.

## 1 Introduction

Tuberculosis (TB), also known as “phthisis” and “white plague,” is a chronic infectious disease caused by *Mycobacterium tuberculosis* (*Mtb*), which endangers human health. According to the Global Tuberculosis Report by the World Health Organization (WHO) in 2022 (1), TB is the second most deadly single infectious disease after coronavirus disease 2019, with 10.6 million new cases of TB worldwide recorded in 2021 and 1.6 million deaths due to TB. Multidrug-resistant tuberculosis (MDR-TB), extensively drug-resistant tuberculosis (XDR-TB), and tuberculosis combined with human immunodeficiency virus (HIV) infection have further increased the global economic burden. Hence, the prevention and control of TB is a major public health issue.

Genomics studies on *Mtb* have shown that the Pro-Glu motif-containing (PE) and Pro-Pro-Glu motif-containing (PPE) gene family is present in pathogenic *Mtb*. Also, 99 PE genes and 69 PPE genes (accounting for ~10% of the coding sequence, encoding 168 proteins in total) are closely related to bacterial virulence (2, 3). The N-terminal sequence of this protein family is relatively conserved, and the C-terminal sequence is highly polymorphic. According to the difference in the C-terminal motif, this protein family can be divided into PPE-PPW (contains the PxxPxxW sequence), PPE-SVP (contains the Gxx-SVPxxW sequence), PPE-MPTR (major polymorphic tandem repeat), and PE-PGRS (polymorphic GC-rich-sequence). The variable C-terminal sequence may be the molecular basis of mutations of the PE-PPE gene or how *Mtb* evades immune attack by the host. In contrast, PGRS or MPTR is absent in rapidly growing non-pathogenic mycobacteria such as *Mycolicibacterium smegmatis* (*Ms*) (4, 5).

Most PE/PPE family proteins are localized in the cell wall and can inhibit macrophage apoptosis (6). This location indirectly enables bacteria to survive and spread in pulmonary macrophages, which is important in immune escape and interaction between the pathogen and host immune cells. Compared with *Mtb* grown *in vitro*, PE11, PE34, PE-PGRS14, PE-PGRS33, PE-PGRS57, and PE-PGRS62 are more abundant in the granuloma tissues of patients with pulmonary TB, and the upregulation trend is statistically significant (7). This observation supports the notion that some PE/PPE proteins enhance the ability of pathogens to survive in the host under unfavorable environmental conditions. PE-PGRS3 mediates adhesion to the type 2 pneumocytes through a unique arginine-rich C-terminal motif. This interaction of PE-PGRS3 with type 2 pneumocytes allows *Mtb* to acquire cardiolipin and phosphatidylinositol for integration into *Mtb* as a source of raw material for phosphate synthesis if phosphate is lacking in the environment in which *Mtb* grows (8). The ability of the protein to capture materials essential for growth and development from the host during crucial processes in TB pathogenesis is thought to be why *Mtb* can survive for a long time in caseous granulomas and foamy macrophages (8).

The Bacillus Calmette–Guerin (BCG) vaccine has been unable to curb the spread of TB in some underdeveloped countries or regions (9). BCG can help stop children from contracting TB, but not adults (10). With the increase in MDR-TB and XDR-TB cases, new TB vaccines with stronger and wider immunity are needed to prevent TB. This review summarizes the role of PE/PPE family proteins and the research progress of related vaccines against TB to provide a reference for further research on PE/PPE family proteins and related vaccines.

## 2 PE/PPE family proteins regulate the immune function of host cells

### 2.1 Regulation of *Mtb* virulence

Secretion of PE/PPE proteins is dependent upon the early secreted antigenic target 6 kDa (ESAT-6) secretion system (ESX) (11). Mutations in PPE38 can block the secretion of the two major ESX-5 substrates, PPE-MPTR and PE-PGRS, thereby increasing the virulence of bacteria and promoting the transmission of hypervirulent strains. Hence, the substrate of ESX-5 appears to be indispensable for attenuating bacterial virulence (12). The highly pathogenic Beijing strain has defective expression of PPE38 and a lack of secretion of the ESX-5 substrate. Knockout of Rv2352c expression can increase the virulence of moderately virulent *Mtb* and manifests as active bacterial growth and apparent inflammatory damage (13). Some studies have found that the ability of PE-PGRS33 knockout mutant (MtbΔ33 strain) to invade macrophages is decreased. Nevertheless, the ability of intracellular replication and immune regulation of this strain has not been observed in a mouse model of TB. The MtbΔ33 strain has shown increased virulence and pathogenicity by aggravating lung tissue damage during chronic infection. PPE27 was expressed in non-pathogenic *Ms* to form Ms-PPE27. Ms-PPE27 and Ms-Vec were injected into mice in a single-cell suspension, respectively, and the colony-forming units (CFUs) in different tissues of the two groups were compared on days 3, 6, and 9. Ms-PPE27 increased the number of bacteria in the lungs, spleen, and liver of mice significantly, and the clearance rate was slow, which prolonged the survival time of Ms *in vivo* (14). The release of lactate dehydrogenase from the supernatant of ANA-1 macrophages infected with PPE27 at 24 h and 48 h was significantly higher than that of Ms-Vec and negative controls. Those results suggest that PPE27 may induce macrophage necrosis, thereby contributing to disease progression (15). *Ms* with overexpression of PE-PGRS19 has a higher tolerance to isoniazid *in vitro*. The former can accelerate the growth rate and survival ability of Ms-Vc and increase the invasion ability and infection rate of Ms-PGRS-19 to macrophages significantly. Those observations indicate that PE-PGRS19 can aggravate bacterial damage to the host and cause toxicity in infected cells (16). In addition, Rv0256c (PPE2) contains a DNA-binding site on the leucine zipper and a functional monopartite nuclear localization signal (NLS) at 473–481 amino acids in the C-terminus of the protein. With the help of the latter, PPE2 can target the nucleus and bind to the gene-regulatory region of the host to manipulate gene transcription, thereby reducing its immune defense function and helping *Mtb* to establish a stable infection *in vivo* (17). As a second messenger, nitric oxide (NO) can stabilize the structure and function of hypoxia-inducible factor (Hif)-1α so that interferon (IFN)-γ can exert an optimal anti-inflammatory effect. If the function of Hif-1α is impaired, even if IFN-γ is maintained at a high level, Hif-1α-deficient macrophages cannot exert a normal killing effect against *Mtb*. PPE2 can block the release of inducible nitric oxide synthase (iNOS) and NO and inhibit the physiological function of Hif-1α competitively because it contains two unique structures and acts as an analog of host transcription factors (18). Meanwhile, PPE2 can also interact with the p67 subunit of reduced nicotinamide adenine dinucleotide phosphate (NADPH) to weaken the activity of the NADPH oxidase system and reduce the content of reactive oxygen species (ROS). These actions are conducive to the continuous growth and reproduction of *Mtb*, leading to higher bacterial load in macrophages and long-term survival of *Mtb* (16, 19).

### 2.2 Interaction with essential trace elements in the body, such as iron and calcium

As a second messenger, calcium [in the form of calcium ions (Ca^2+^)] plays a key part in cellular signal transduction and maintenance of homeostasis and is also indispensable for the development and acidification of phagosomes. *Mtb* can inhibit the maturation of phagosomes by chelating intracellular Ca^2+^ (20). Calmodulin (CAM) is a calcium-binding protein that has an important adaptor role in Ca^2+^-mediated signaling pathways in various cellular responses. In bacteria, calmodulin-like proteins (CAMLPs) have a high degree of domain homology with eukaryotic CAM. The protein encoded by Rv1211 is a phosphodiesterase and NAD-kinase CAMLP. During *Mtb* infection, CAMLP can inhibit the fusion of phagosomes and lysosomes by binding Ca^2+^ and reducing the concentration of free Ca^2+^ in macrophages, thereby weakening the pernicious effect of immune cells on pathogens and promoting the intracellular survival of *Mtb* indirectly (21, 22). Rv1818c (PE-PGRS33) and Rv3653 (PE-PGRS61) also have Ca^2+^-binding properties. The CFU of THP-1 cells infected with *Ms* expressing Rv1818c and Rv3653 was shown to be significantly higher than that of Ms-Vc at 24 h, 48 h, or 72 h. Increased counts at each time point correlated with the downregulation of iNOS expression (a key determinant of intracellular load in the host). Interleukin (IL)-10 is an anti-inflammatory inhibitory factor that negatively regulates the defense function of immune cells (23), the IL-10 content in the supernatant of THP-1 cells infected with the two strains was increased significantly. After Ca^2+^ depletion with the chelator ethylene glycol-bis (β-aminoethyl ether)-*N,N,N′,N′*-tetraacetic acid, this response was abolished completely (22). Those results indicate that Ms-1818c and Ms-3653 increase IL-10 content in a Ca^2+^-dependent manner to improve their viability (22).

Iron ions are essential trace elements for the human body. Studies have found that cluster of differentiation (CD)4^+^ T cells increase iron demand significantly after activation, while iron deficiency impairs the epigenetic regulation of T-helper (Th)17 cells and hinders the proliferation and differentiation of CD4^+^ T cells and CD8^+^ T cells (24). *Mtb* competes with host cells to uptake iron from the environment through siderophore-mediated iron acquisition (SMIA) and heme iron acquisition (HIA). SMIA captures iron from lactoferrin and transferrin. HIA can obtain iron ions from hemoglobin (which is also closely related to the pathogenic process of *Mtb*). Normal *Mtb* utilizes the low concentration of heme in the medium efficiently, and if siderophore synthesis was blocked or dysfunctional, bacteria grew poorly or stopped growing in an iron-rich 7H9 medium and returned to average growth after the addition of extra siderophore-like molecules (25, 26). Deleting PPE37 resulted in a Mtb△PPE37-deficient strain, which grew poorly in the original medium with almost unchanged concentrations of heme and iron. Approximately 200 times the heme concentration in the original medium was required to achieve similar growth to that of parental *Mtb*. BCG is a PPE37 mutant strain, and survival in a low-iron environment was consistent with that of wild-type (WT) *Mtb* only if the gene encoding functional PPE37 was added to BCG. Those results suggest that PPE37 is crucial in the pathological process of iron interception through HIA, which may be related to the reduced permeability of bacterial cell walls to heme, but the specific mechanism warrants further exploration (26).

### 2.3 Regulation of apoptosis, pyroptosis, and autophagy in the host

During *Mtb* infection, PE/PPE family proteins can regulate various cell death pathways, such as apoptosis and pyroptosis (27). The mitochondria are important organelles involved in apoptosis, so proteins targeting the mitochondria of host cells may play a part in regulating infection immunity and apoptotic pathways (28). The C-terminal sequence of PE6 (Rv0335c) contains two homology domains similar to BH3 in the proapoptotic protein B-cell lymphoma-2 (Bcl-2). Apoptosis-related proteins activate each other through BH3 to promote apoptosis on the one hand, and PE6 can enhance TLR4 expression and upregulate the level of tumor necrosis factor (TNF)-α on the other hand, because PE6 contains a BH3-like domain at its C-terminus, and it can also take advantage of the mitochondrial processing peptidase activity of this domain to target the mitochondria to cleave its signal peptide sequence, resulting in increasing cytosolic levels of cytochrome C (CytC) and intracellular Ca^2+^ loading, inducing caspase-mediated apoptosis of macrophages and promoting the long-term survival of *Mtb*. However, no obvious Ca^2+^ influx was observed in Rv0335cΔC-ter-infected cells, compared with intact PE6, and the content of caspase-3/7/9 and the proportion of apoptotic cells were significantly decreased in Rv0335cΔC-ter-infected cells (29). Sharma et al. treated RAW264.7 cells with PE6 (5 µg/mL, 7.5 µg/mL, or 10 µg/mL) for 24 h and detected apoptosis by flow cytometry. They found that PE6 at 5 µg/mL could promote macrophage apoptosis in a concentration-dependent manner. Meanwhile, protein assays showed that PE6 increased the secretion of proapoptotic proteins Bax and CytC and activated caspase-3. Higher levels of unfolded proteins (UPs) such as C/EBP homologous protein (CHOP), p-protein kinase R (PKR)-like endoplasmic reticulum kinase (PERK), and phosphorylated (p)-eukaryotic initiation factor-2α (eIF2α) were also detected, indicating that PE6 induces the production of a large number of proteins of ER stress-related responses in macrophages, thereby causing macrophage apoptosis (30). After THP-1 cells had been infected with PPE10 (Rv0442c) for 6 h or 24 h, the expression of caspase-3, 7, and 8 in the host cells was decreased. Reverse transcription-polymerase chain reaction showed that Bax transcription was decreased significantly, suggesting that apoptosis was reduced and that PPE10 could inhibit the apoptosis of host cells and promote *Mtb* survival indirectly (31). Persistent ER stress can trigger a regulation cascade to initiate apoptosis signals (32). PE-PGRS1 downregulates the expression of the ER stress-induced markers C/EBP homologous protein (CHOP), phosphorylated (p)-eukaryotic initiation factor-2α (eIF2α), and p-protein kinase R (PKR)-like endoplasmic reticulum kinase (PERK), thereby inhibiting the intracellular stress of THP-1 cells induced by *Ms*. These actions reduce the content of caspase-3/9 and permit alternative splicing of Bax to inhibit apoptosis, which is conducive to the survival, reproduction, and pathogenesis of bacteria in macrophages (33).

Apoptosis is considered to be a relatively “safe” form of cell death. Simultaneously, pyroptosis is accompanied by a strong inflammatory response, which is caused by the expansion and rupture of the plasma membrane due to intracellular and extracellular pathogens or toxins, and results in the release of many cytokines and bacteria (34). Therefore, it has been speculated that pyroptosis promotes the dissemination of *Mtb in vivo* to a certain extent, leading to the chronic trend of TB. Peroxisome proliferator-activated receptor (PPAR)γ binds competitively to the p65/p50 subunit of nuclear factor-kappa B (NF-κB) to inhibit the function of mononuclear macrophages and expression of proinflammatory factors. In one study, PPARγ transcription in Ms-PPE60-infected cells was inhibited, whereas the expression of the proinflammatory cytokines IL-1β, IL-6, and IL-12 was upregulated. Mitochondrial fusion protein (Mfn)2 is located in the outer membrane of the mitochondria and has anti-apoptotic activity. Quantitative detection of Mfn2 showed that PPE60 did not change the Mfn2 level, indicating that the integrity of the mitochondrial membrane was unaffected. However, messenger (m)RNA expression of important pyroptosis molecules such as caspase-1/4, NOD-, LRR-, and pyrin domain-containing protein 3 (NLRP3) and gasdermin D (GSDMD) was increased significantly, suggesting that PPE60 induced macrophage pyroptosis to a greater extent in this form of cell death (35). Compared with Ms-Vec, Ms-PPE13 enhanced IL-1β secretion and cleaved GSDMD into more GSDMD-NT (p30) by activating NLRP3 and caspase-1, which translocated to the cell membrane to form a pore (10–15 nm) and led to leakage from the plasma membrane. Finally, progression to GSDMD-mediated pyroptosis was observed. Interestingly, Ms-Vec induced 10 times more GSDMD expression than Ms-PPE13 after 48 h of culture, but the amount of IL-1β in the supernatant that seeped through GSDMD wells in the Ms-PPE13 group was higher than that in Ms-Vec group. Those results suggest that PPE13 may also cleave other members of the gasdermin family (e.g., GSDMA, GSDMB, GSDME), which are involved in cell membrane pore formation and pyroptosis (36). In addition, PE-PGRS19 is a novel agonist of the non-canonical pyroptosis pathway (caspase-11-GSDMD-Il-1β/18) in *Mtb*, which leads to pyroptosis by activating caspase-11 and inducing GSDMD cleavage to the p25 fragment (15).

Autophagy is a process of cellular self-degradation. Autophagy is essential to innate immunity and adaptive immunity. It plays a vital part in the resistance to bacterial infection and the clearance of intracellular pathogens (37). High-throughput screening of a transposable sequence mutation library of *Mtb* for loss of function revealed that *Mtb* contained 16 inhibitory autophagy-related genes, of which six encoded PE-PPE family proteins (Rv1068c, Rv1087, Rv1651c, Rv2471, Rv2770c, Rv3136) (38). PE-PGRS47 has been reported to prevent the acidification and maturation of phagosomes and the association of autophagosomes with lysosomes, thereby increasing the virulence and resistance to the adverse intracellular environment of *Mtb*. p62 expression in WT *Mtb*-infected cells was shown to be higher than that in △PE-PGRS47 mutant cells, which could be restored to the same level as that in WT *Mtb*-infected cells (39). Strong et al. demonstrated that PE-PGRS20 and PE-PGRS47 inhibited the transition of the Unc-51-like kinase (ULK1) complex to autophagosomes by interacting with the Rab1A protein, resulting in reduced phosphorylation of ULK1 at the mammalian target of rapamycin (mTOR)C1 and negative autophagy. Compared with *Mtb* infection in WT cells, RAW264.7 cells infected with △PE-PGRS20 or △PE-PGRS47 had increased autophagy-related gene 5 (Atg5) content and decreased p62 content, indicating that autophagy was increased in cells infected with mutant strains. However, PE-PGRS expression inhibited the activation of the autophagy pathway and intracellular anti-infective efficacy against *Mtb* (40).

### 2.4 Regulation of cellular immunity

**Figure 1:**
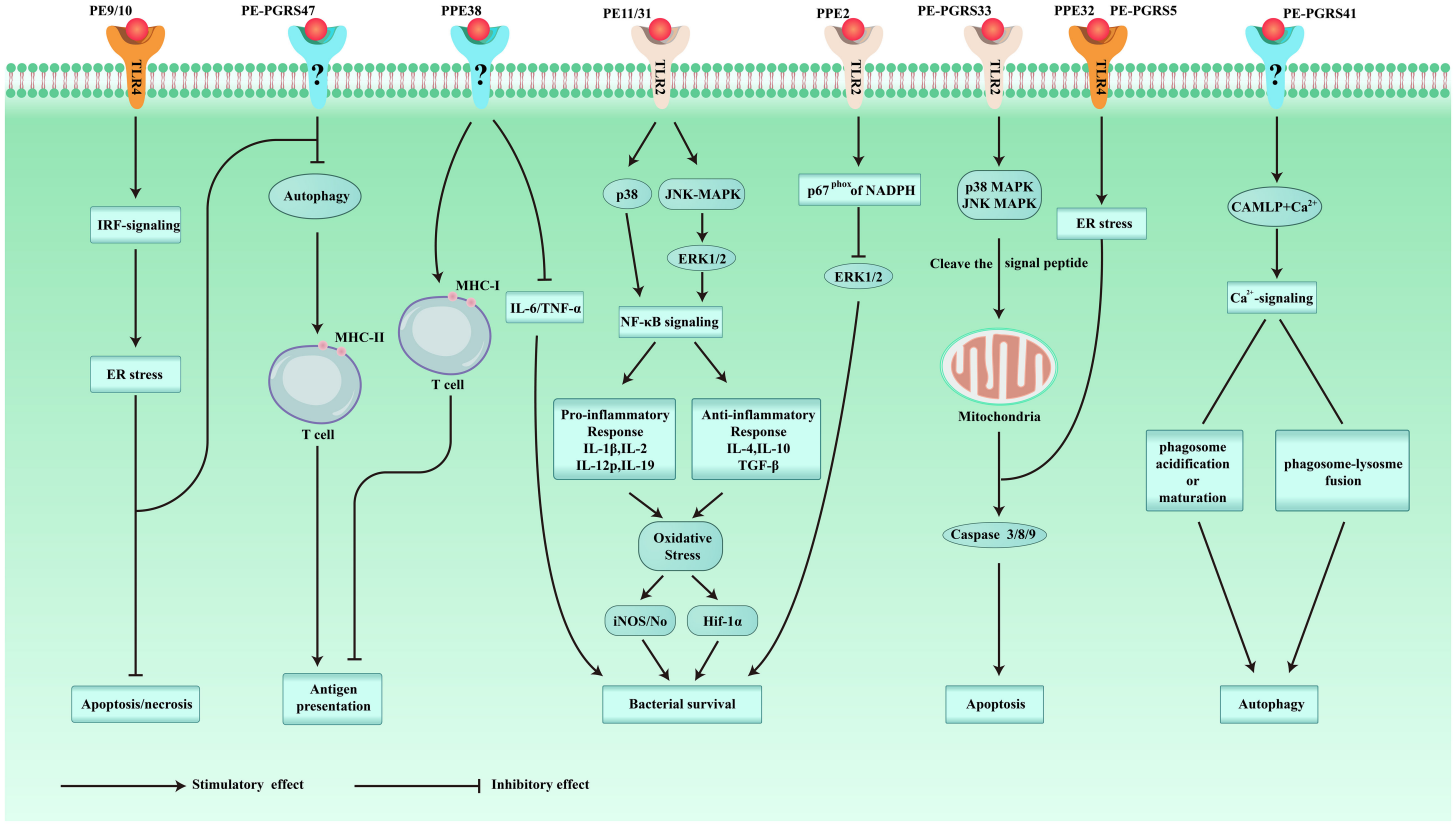
Schematic representation of some of the immunomodulatory roles played by PE/PPE proteins.

**Figure 2:**
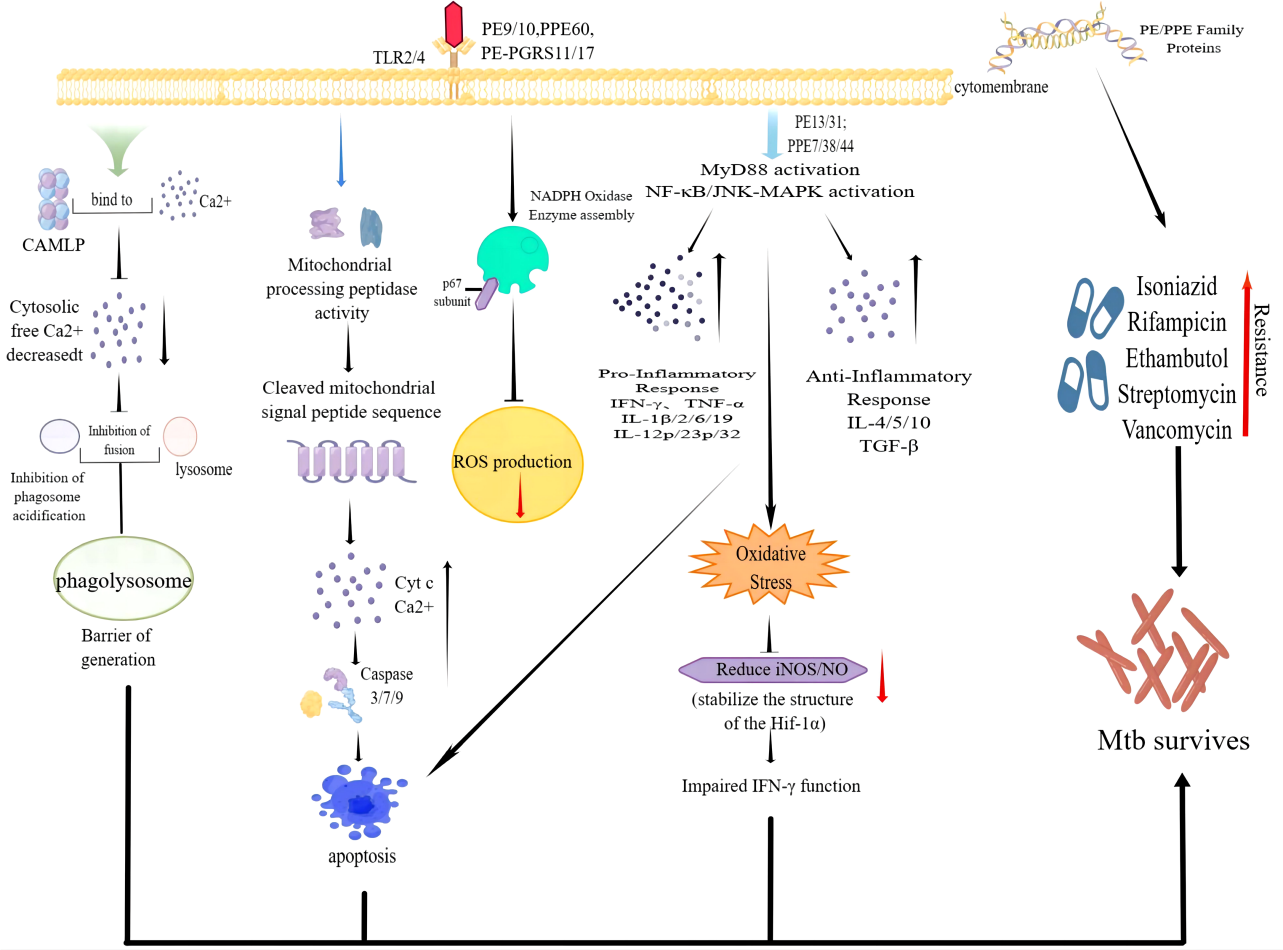
Some PE/PPE family proteins function intracellular to promote the survival of Mtb.

Cellular immunity plays a significant part in the protective immunity against *Mtb*. PE/PPE proteins can induce T cells to produce cellular immunity. The crucial prerequisite for this immune response is the activation of antigen-presenting cells (APCs) and the effective presentation of antigenic peptides. Dendritic cells (DCs) are crucial for antigen presentation. Initiation of the Th1-type immune response is essential for controlling *Mtb* replication within the host cells. Choi et al. isolated PPE39 from a virulent Beijing strain of *Mtb*. They treated DCs with PPE39 and found that expression of the major histocompatibility complex (MHC) type-I/II molecules CD80 and CD86 on the surface of DCs was enhanced. Those data indicated that PPE39 could induce DC maturation and that DCs treated with PPE39 could promote the proliferation of CD4^+^ T cells. Meanwhile, the expression of the Th1-related transcription factor T-bet was increased, but the contents of Th2-related molecules and proteins were not increased (41). TLRs are the only known host cell receptors for PE/PPE proteins (42). Their interaction regulates the release of proinflammatory/anti-inflammatory cytokines by activating NF-κB and c-Jun N-terminal kinase-mitogen-activated protein kinase (JNK-MAPK) signaling pathways. Some studies have found that PE-PGRS11 (Rv0754) and PE-PGRS17 (Rv0978c) can induce the maturation and activation of human DCs and stimulate the secretion of proinflammatory cytokines by recognizing TLR2 (43). PE-PGRS33 and its PE domain were found to promote IFN-γ secretion and the proliferation of CD4^+^ T cells and CD8^+^ T cells in BALB/c mice and latent *Mtb* infection (44). PE31 induced the expression of the anti-inflammatory cytokine IL-10 by activating the downstream pathway of NF-κB. At 48 h after infection, IL-10 expression was increased significantly, whereas transcription of IL-6 and IL-12 was decreased significantly, and the presentation of activated caspase-3 protein fell. Those data suggest that PE31 may reduce macrophage apoptosis (45). IL-10 expression has been found to be increased in macrophages infected with Ms-PE-PGRS41, and regulation of IL-6 is similar to that by PE31 (46). It has been found that IL-10 inhibits phagosome maturation in *Mtb*-infected human macrophages, leading to a reduction in the amount of proinflammatory cytokines (47). This observation suggests that specific PE/PPE family proteins can enhance *Mtb* resistance within the host cells by inhibiting the release of proinflammatory cytokines. PPE65 can trigger signaling pathways that secrete proinflammatory factors by binding to the leucine-rich repeats (LRR) domain of TLR2, such as the interleukin-1 receptor-associated kinase 3 (IRAK3) cascade, to stimulate NO release and upregulate the expression of IL-6 and TNF-α (48). As an agonist of TLR4, PPE39 can also activate MAPK and NF-κB pathways to trigger DCs (49), induce the maturation and activation of DCs, promote the polarization of Th1 cells, and control the growth of intracellular *Mtb*. In conclusion, PE/PPE family proteins regulate *Mtb* survival in host cells by binding to TLRs to activate cell signaling pathways and regulate the secretion of inflammatory factors. PPE7 can prolong the survival of *Mtb* in macrophages by activating MAPK and NF-κB signaling pathways and regulating the extracellular signal-regulated kinase (ERK)–p38–NF-κB axis. In one study, the expression of TNF-α, IL-6, and IL-1β in THP-1 cells was increased significantly within 48 h after infection, whereas secretion of the anti-inflammatory factor IL-10 was inhibited. Due to the release of proinflammatory factors, the colony load of Ms-PPE7 in the organs of infected mice was increased significantly, and they all showed severe tissue damage caused by a rapid inflammatory response. Microscopy revealed thickening of the alveolar septum with exudation of red blood cells, infiltration by many inflammatory cells in the lungs and spleen, and punctate necrosis in the liver. In addition, Ms-PPE7 could also resist the threats of a high concentration of lysozyme (2.5 g/mL) and a more acidic medium (pH = 3) (50). After combining with herpesvirus-associated ubiquitin-specific protease (HAUSP), PE-PGRS38 could regulate cytokine levels in mouse bone marrow-derived macrophages (BMDMs) by inhibiting the deubiquitination of tumor necrosis factor receptor-associated factor (TRAF) 6 by HAUSP. Through downregulating the expression of TNF-α, IL-1β, IL-6, and IL-10, the inflammatory response *in vivo* could be balanced to a suitable environment for *Mtb* survival, thereby increasing the duration of intracellular survival and potency of bacteria. Interestingly, PE-PGRS38 was also observed to reduce the hyperinflammation caused by a high bacterial load, which seems to be a “beneficial” phenomenon for the host (51). The **Figures 1** and **2** briefly shows the immunological mechanism of PE/PPE proteins. We summarize the subcellular localization of the different PE/PPE proteins and their mechanisms of action with the host in **Table 1**.

**Table 1:** Subcellular localization and mechanism of action of PE/PPE family proteins in *Mycobacterium tuberculosis*.

Protein	Subcellular localization	Effect on immune response	References
PE4	Cytomembrane	Expresses at the chronic stages and enhances *Mtb* survival during hypoxia	(52)
PE9/PE10	Cytomembrane	Interacts with TLR4 and induces apoptosis via IRF3 signaling	(53)
PE11	Cytoderm	Involvement in bacterial cell wall remodeling by modifying fatty acids	(54, 55)
PE13	Cytomembrane	Inhibits apoptosis and enhances stress resistance capacity of *Mtb*	(52)
PE16	Cytomembrane	Regulates intracellular triglyceride levels	(56)
PE20	Cytomembrane	Involved in magnesium ion transport in *Mycobacterium tuberculosis*; promotes cell division and metabolism	(57)
PE25/PE41	Cytomembrane	Induces macrophage necrosis to facilitate the dissemination of pathogens	(58)
PE27	Cytomembrane	Activates DC and the expression of co-stimulatory molecules and upregulates the production of proinflammatory cytokines via MAPK–NF-κB signaling	(59)
PE31	Cytomembrane	Attenuates host cell apoptosis; Up-regulates production of anti-inflammatory cytokines and down-regulates production of proinflammatory cytokine	(45)
PPE2	Cytomembrane	Inhibits NO production by downregulating the expression of the iNOS gene; contains SH3 domain which enables its binding to p67phox and downregulates ROS levels	(19, 60)
PPE13	Cytomembrane	Activates NLRP3 inflammasome and induces cleavage of caspase-1 and secretion of IL-1β	(36)
PPE18	Cytoderm	Upregulation of IL-10 and inhibition of IL-12/TNF-α production in macrophages; prolongs the survival time of bacteria in macrophages	(81)
PPE25	Cytomembrane	Up-regulates production of proinflammatory cytokines via p38-MAPK-ERK-NF-κB signaling and induces host cell necrosis	(61)
PPE26	Cytomembrane	Interacts with TLR2 and enhances the expression of co-stimulatory molecules along with CXCR3 and CD4 T-cell responses; upregulates the production of proinflammatory cytokines via p38–MAPK–ERK–NF-κB signaling and induces host cell necrosis	(61, 62)
PPE32	Cytoderm	Promotes ER stress-mediated apoptosis involving caspase 3/9 activation	(63)
PPE36	Cytomembrane	Involved in heme transport across the membrane	(64)
PPE37	Cytomembrane	Encodes siderophore associated with iron uptake; reduces the content of IL-6/TNF-α	(26)
PPE38	Cytomembrane	Induces macrophages to secrete IL-6/TNF-α; downregulates MHC-I antigen presentation	(65)
PPE57	Cytomembrane	Interacts with TLR2 and enhances the expression of co-stimulatory molecules and establishes cross-talk with p38–ERK–NF-κB signaling	(66)
PPE60	Cytoderm	Interacts with TLR2 and activates maturation of DC and expression of co-stimulatory molecules (CD80, CD86, MHC-I/II); upregulates the production of proinflammatory cytokines; induces pyroptosis via NF-κB signaling; enhances the stress resistance capacity of *Mtb*	(35, 67)
PPE62	Cytomembrane	Heme-iron acquisition and growth of pathogen	(64)
PPE68	Cytomembrane/cytoderm	Regulates antigen release ESX-1 gating channel; promotes bacterial survival in the host; induces IFN-γ secreting CD4^+^ T cells and host cell necrosis	(68, 69)
PE-PGRS3	Cytoderm	Promotes adhesion to macrophages and alveolar epithelial cells; increases persistence in host tissues	(8, 70)
PE-PGRS5	Cytoderm	Induces caspase-3/8/9-mediated apoptosis and is UPR/TLR4-dependent	(71)
PE-PGRS11	Cytomembrane	Encodes functional phosphoglycerate mutase; enhances resistance to H_2_O_2_-induced oxidative stresses thanks to the anti-apoptotic signals triggered by the TLR2-dependent activation of COX-2/Bcl-2 expression	(72)
PE-PGRS17	Cytoderm	Interacts with TLR2 and activates maturation of DC and stimulation of co-stimulatory molecules and upregulates the production of proinflammatory cytokines via MAPK-NF-κB pathway; induces host cell death with secretion of TNF-α via the Erk kinase pathway	(43, 73)
PE-PGRS18	Cytoderm	Promotes cell apoptosis; induces IL-12 and inhibits IL-6, IL-1β, and IL-10	(74)
PE-PGRS30	Cytoderm	Blocks phagosome maturation to enhance *Mtb* intracellular survival	(75, 76)
PE-PGRS33	Cytoderm	Induces necrosis and apoptosis; increases IL-10 and decreases IL-12/TNF-α in macrophage; induces inflammation in a TLR2-dependent mechanism; promotes entry in macrophages via the TLR2/CR-3 inside-out signaling pathway	(14, 77)
PE-PGRS41	Cytoderm	Promotes anti-inflammatory response; inhibits host cell apoptosis and autophagy	(78)
PE-PGRS47	Cytoderm	Blocks autophagy and phagosome acidification; inhibits MHC-II antigen presentation which suppresses *Mtb*-specific CD4^+^ T-cell responses	(39)
PE-PGRS61	Cytoderm	Binding of calcium to PE-PGRS61 increases affinity toward TLR2 and upregulates anti-inflammatory cytokine IL-10	(22)
PE-PGRS62	Cytoderm	Inhibits phagosome–lysosome maturation; reduces the production of NO in macrophages	(6)

### 2.5 Involvement in humoral immunity

B cells are important components of tuberculous granulomas and regulate the inflammatory process by secreting antibodies and IL-10 (79). However, *Mtb* is an intracellular pathogen, so the classical view is that CD4^+^ T cells and Th1-type cytokines are mainly responsible for the components that have an anti-TB effect *in vivo*. Hence, few reports have focused on the changes in B cells during *Mtb* infection (80). This lack of research has led to a long underestimated contribution of humoral immunity to the control of *Mtb* infection.

Some studies have found that PPE18 participates in cellular immunity but also limits humoral immunity to a certain extent, which provides survival advantages for *Mtb*. PPE18 interferes with the uptake and processing of antigens by APCs in a dose-dependent manner, which affects the formation of MHC–antigen peptide complexes, resulting in impaired activation of CD4^+^ T cells and significant reduction in the IL-2 level. If peripheral blood mononuclear cells (PBMCs) are treated with PPE18, the response of lymphocytes to purified protein derivative (PPD) is weak. Three days after infecting mice with Ms-PPE18, flow cytometric analysis showed that compared with Ms-pVV16 infection, PPE18 increased the percentage of immature B cells in mice (26.14%) and that the dark zone/light zone (DZ/LZ) ratio at the germinal center increased. The number of B cells in the bright area was low, suggesting that B-cell activation had been impaired. Continued infection of mice revealed low levels of mouse-specific immunoglobulin (Ig)G and IgM antibodies on days 11 and 21. Those results indicate that PPE18 interferes with the humoral immune response by inhibiting the maturation and activation of B cells and antibody production (81). B cells play an important role in regulating host response and curbing *M. tuberculosis* infection. Impairment of B cells enhances the susceptibility of mice to tuberculosis, and B lymphocyte-deficient mice challenged with *Mtb* exhibit higher visceral bacterial loads (82).

## 3 Novel vaccines based on PE/PPE family proteins against TB

BCG is one of the most widely administered vaccines worldwide, with approximately 100 million newborns receiving BCG each year (83). BCG vaccination within 1 week of birth can protect 73% of newborns against tuberculous meningitis and 77% against miliary TB (84). BCG also improves the innate immune response to pathogenic microorganisms other than *Mtb*, such as *Candida albicans* and *Staphylococcus aureus*, indicating that BCG could induce non-specific cross-protection against pathogens unrelated to *Mtb* in young children (85). The novel coronavirus infection that began in late 2019 was a catastrophic event for global public health, which has worsened the TB prevention and control situation. *Mtb* infection could increase host susceptibility to severe acute respiratory syndrome coronavirus 2 (SARS-CoV-2) infections (86). Coronavirus disease 2019 (COVID-19) co-infection with TB can cause a large number of inflammatory cell infiltration in the lung; further enhance the immune response of the injured site; produce excessive cytokines like IL-1, IL-6, IL-10, IL-18, and IFN-α; and promote cytokine storms that lead to multiple organ dysfunction, resulting in a higher risk of death than a single pathogen (87, 88). However, it is surprising that some studies have shown that BCG has a certain degree of non-specific cross-protection against SARS-CoV-2. Previous studies have shown that BCG vaccination protects against tuberculosis, herpes, and influenza virus infections and reduces their morbidity and mortality (89). This may be due to heterologous immunity by antigen-independent activation of B and T cells and reprogramming of innate immune cells—the effect exerted by training immunity (90). BCG can regulate the response of lymphocytes to secondary infection by stimulating CD4^+^ and CD8^+^ T cells against untargeted antigens, thereby increasing the resistance of non-specific proinflammatory cytokines IL-1β and IL-6 to pathogens (85) and promoting the immune response of innate immune cells including monocytes, natural killer cells, and alveolar macrophages, leading to increasing the host resistance to infection with a variety of pathogens, especially SARS-CoV-2 (86, 91), so BCG can prevent or reduce SARS-CoV-2 infection, inhibit virus replication, reduce viral load, and further alleviate inflammatory damage and clinical symptoms, especially in children vaccinated with BCG (92, 93). Counoupas et al. (94) proposed the use of an interesting combination of BCG and a trimeric SARS-CoV-2 spike protein antigen (BCG : CoVac), which induces the generation of specific T cells in mice that promote the production of Th1-type cytokines and high-titer IgG neutralizing antibodies. A single dose of BCG : CoVac completely eliminated the symptoms and significantly reduced inflammation in challenged animals. Surprisingly, no viral load was detected in the lungs of any of these challenged animals. A randomized, double-blind, placebo-controlled trial conducted in the USA showed that multi-dose BCG vaccine was effective in preventing and reducing the severity of infection in patients with type 1 diabetes—a high-risk factor for SARS-CoV-2 infection—who had not previously received either BCG or COVID-19 vaccines (95). In another phase III, multicenter, double-blind trial in which 301 volunteers aged >50 were randomized (1:1) to BCG or placebo, approximately 5% of the participants in the BCG group had positive antibodies against SARS-CoV-2, with a 68% lower risk for COVID-19, compared with the placebo group (96). Although the WHO has not recommended the use of BCG as a means to prevent or treat COVID-19, it is anticipated that BCG will be considered an ideal measure to fight COVID-19 for developing and underdeveloped countries that lack treatment. However, the efficacy of BCG vaccination in adults is not high. The individual variation in the efficacy of BCG against TB in adults has been reported to range from 0% to 80% (97). Because of regional and ethnic differences, the average percentage protection of BCG against TB in children in some areas is only 52% (98). In addition, BCG is a live attenuated vaccine, so it is unsuitable for people who are immunosuppressed or immunodeficient. BCG vaccination in HIV-infected patients is prone to strain mutation or BCG-disseminated disease (99). Therefore, there is an urgent need to develop safer and more efficacious new vaccines to prevent and treat *Mtb* infection.

The PE/PPE vaccine of *Mtb* is a component vaccine. It is made by screening and purifying the immunogenic PE/PPE protein, combining it with other types of antigens, and aligning it with adjuvants or carriers. This type of vaccine has stability and safety and can fuse various protective antigens selectively to aid expression, thereby improving its specificity and the level of immune response *in vivo*. Most PE/PPE vaccines are used as booster vaccines. They are designed to enhance the immune response and prolong the duration of resistant protection after vaccination with a primary vaccine such as BCG. The section below reviews recent vaccines against TB based on PE/PPE family proteins as antigen targets. **Table 2** presents the immunoprotective effects of fusion proteins or vaccines incorporating members of the PE/PPE family proteins.

**Table 2:** Comparison of PE/PPE family protein-associated TB vaccines.

Vaccine	Composition	Advantage	Deficiency
ChAdOx1-PPE15	PPE15, chimpanzee adenovirus vector	It can effectively clear *Mtb* and enhance the immune efficacy of BCG	The results varied depending on the mouse lineage.
M72:AS01	Fusion protein of PPE18-Rv0125, AS01 adjuvant. It is currently in phase IIb clinical trials.	The Th1 response is strong and prevents LTBI from progressing to the active stage. The protective effect of M72 was better than that of BCG and significantly prolonged the survival time of the challenged animals. Combined treatment with BCG could reverse the lung injury caused by tuberculosis.	The bacterial load in the lungs of infected animals could not be significantly reduced. There are regional and ethnic differences, which need to be further verified by clinical trials.
ACP	Fusion protein of Ag85B-CFP21-PPE18	The specific antibody increased significantly and reduced the CFU and inflammatory damage in the lung.	It did not improve the immune effect of BCG.
INP-0288-1196-0125	The fusion protein Rv0288-Rv1196 (PPE18)-Rv0125 was expressed on INP.	The effect within a certain range increased with the increase in immunization times.	The experimental animals were single.
ID93:GLA-SE	The fusion protein of Rv1813-Rv3620-Rv3629-PPE42, GLA-SE adjuvant. It is currently in phase IIa clinical trials.	Various immune pathways can improve the protective efficacy of BCG, improve drug efficacy, and prevent *Mtb* recurrence. It can effectively reduce the mortality of tuberculosis in experimental animals.	It is necessary to further compare the differences between different vaccination methods to find the best immunization route.
rBCG::Ag85B-EAST-6-PPE42	The fusion protein of Ag85B-EAST-6-PPE42 was expressed in BCG.	A stronger specific response was induced than that of BCG.	Duration and immune memory are unknown.
Tri-Fu64	The fusion protein of PPE42-Rv1793-Rv2628	It can significantly reduce the bacterial load in the body and improve the ability of anti-*Mtb* infection.	The protective effect was single, and the combination with MPL or DDA could reduce the body weight of mice.
HPERC	The fusion protein of Rv2031c-EAST-6-PPE44, resiquimod adjuvant	CD4^+^ T-cell response and humoral immunity are obvious while limiting inflammatory damage caused by hyperimmunity.	No corresponding clinical human trial data were available.
Tetrafu56	The fusion protein of Esp-C-TB10.4-PPE57-Hsp-X	A multiphase therapeutic vaccine that produces significant immunity against active pulmonary tuberculosis infection *in vitro*	*In-vivo* experiments have not been carried out. It was not effective in preventing *Mtb* in healthy adults.
A3-len	The fusion protein of Ag85B-PPE57 was expressed in the lentivirus vector.	It reduced the number of bacteria in the lung and spleen, attenuated lung lesions, protected against *Mtb* damage, and restored the body weight of the infected mice.	CD8^+^ T cells were decreased.
A39	Rv2029c was added to A3-len.	It promoted T-cell polarization to Th1 and inhibited bacterial reproduction in the organs.	A variety of animal models are needed for further verification.
rBCG::PPE68	PPE68 was expressed in BCG.	Th1 response was superior to BCG and maintained a high level of humoral immunity.	It still needs to be verified by repeated experiments.
rLmMtb9Ag	Nine antigens including PPE68 were fused in the *Listeria* vector.	Hypervirulent *Mtb* infection can be antagonized in guinea pigs without BCG primary vaccination.	The immunodominant epitope P9 peptide of PPE68 requires further purification.
rBCG::PE-MPT64	The fusion protein of the MPT64-PE segment of PE-PGRS33 was expressed in BCG.	The PE segment targets the delivery of antigenic peptides, and the PE-PGRS antibody inhibits *Mtb* entry into macrophages with better protective efficiency than BCG and can be used for LTBI.	The experiment is still in the preliminary stage and needs further study.

### 3.1 PPE15-related vaccines

PPE15 is involved in the lipid accumulation of latent *Mtb* and plays an important part in stabilizing the lipid synthesis, metabolism, and stress state of *Mtb* (100). Four types of vector vaccines containing PE/PPE proteins have been prepared by expressing PE3, PE12, PPE15, and PPE51 on chimpanzee adenovirus vector ChAdOx1, respectively. Mice were given a single intranasal vaccination. Four weeks later, mice were challenged with an *Mtb*-containing aerosol. The CFU count showed that the vaccine expressing PPE15 and PPE51 could reduce the bacterial load in the lungs and spleen significantly. The degree of inhibition of the PPE15 vaccine was similar to that of BCG, but the two other vaccines (PE3 and PE12) did not control the growth or reproduction of bacteria in organs. Among the vaccines stated above, only the vaccine expressing PPE15 could improve the protective efficacy of BCG in primary immunization, promote the proliferation of CD4^+^ T cells and CD8^+^ T cells induced by BCG, and enhance the clearance ability of BCG against *Mtb* and its protective effect in mice. However, this effect was statistically significant only in C57BL/6 mice, and not in BALB/c mice, and the combination of the four vaccines did not produce an additive protective effect (101). Recently, Xu et al. purified PPE15 and found that it had strong antigenicity and could react specifically with the serum of patients suffering from TB but not with the serum of patients with pneumonia or healthy adults (102). Although few studies have been done on PPE15, those results suggest that PPE15 is a promising antigen target for developing vaccine candidates against TB, but the specific immune mechanism merits further exploration.

### 3.2 PPE18-related vaccines

#### 3.2.1 M72:AS01

Among the vaccines designed to target PPE18, the most advanced is M72:AS01. M72 is a vaccine based on recombinant protein subunits consisting of Mtb39A (Rv0125) and PPE18 (Rv1196). PPE18 is associated with the cell wall of *Mtb* and is an important virulence factor. The MtBΔ18 recombinant strain can protect mice from *Mtb* infection, reduce inflammatory damage, and increase the number of infected mice who survive (103). AS01 is a liposome vaccine adjuvant containing two immunostimulants: lipid monophosphoryl A (MPL) and saponin QS-21 (104). M72:AS01 could generate a comprehensive and robust immune response, resulting in the elicitation of strong IFN-γ and Ab responses and a strong CD8 response directed against the Mtb32 epitope. The protective effect of M72 was better than that of BCG in aerosol-challenged mice and guinea pigs by observing the signs and surviving numbers of mice and guinea pigs challenged with virulent *Mtb* strains at different time periods (0–70 weeks), and the survival time of mice and guinea pigs injected with M72 vaccine is 1 year longer than that of BCG alone (105). Surprisingly, M72 delivered by the coadministration with BCG vaccination significantly improved the survival of these animals compared with BCG alone, with some animals still alive and healthy in their appearance at >100 weeks post-aerosol challenge in the more stringent guinea pig disease model. M72 can improve the ability of BCG to reconstruct the airway and limit the progression of pulmonary consolidation caused by *M. tuberculosis* and promote the regression of lung tissue lesions (106). The cynomolgus monkey is an ideal non-human primate model for tuberculosis vaccine research (107). In another study, it was confirmed that M72 had good safety in cymophagus monkeys, with no body weight loss or abnormal inflammatory indicators such as erythrocyte sedimentation rate before challenge. The combined use of BCG and M72 induced a potent anti-tuberculosis cytokine profile in cynomolgus monkeys, mainly IFN-γ, TNF-α, IL-2, and IL-6, which enabled the challenge animals to achieve long-term survival and reverse the outcome of tuberculosis progression (108). Two doses of the vaccine could induce solid and durable immune responses, with a high frequency of M72-specific CD4^+^ T cells and significant secretion of Th1 cell-related factors. The main adverse reactions appear to be redness and swelling at the vaccination site, but severe safety events have not been observed (109). Phase II clinical trials conducted in India have shown that M72 is well tolerated and immunogenic in HIV-positive populations (110). Compared with several common subunit vaccines that have entered clinical trials (H1, H56, ID93, MVA85, eras-402), M72:AS01 elicited the highest levels of Th1 cytokines and memory CD4^+^ T-cell responses (111), which also highlights the advantages of M72 as a novel vaccine against TB. Analyses of a more extensive phase IIb randomized placebo-controlled trial evaluating M72:AS01 in 3,573 adult volunteers recruited in South Africa, Zambi, nd Kenya found that M72:AS01 protected progression to active pulmonary TB for 3 years in HIV-negative patients with latent tuberculosis infection (LTBI). The percent protection was ~54%, and there were no apparent safety problems, which met the requirements of WHO for new vaccines against TB (112). However, due to regional and ethnic differences, larger and longer trials in broader populations are needed to confirm those results. In addition, the C-terminal domain of PPE18 has many gene polymorphisms and has a high frequency of mutation that changes the polypeptide sequence of the corresponding epitope region. The mutation types are mostly single-nucleotide polymorphisms and frameshifts, which may reduce the immune protection induced by M72 to varying degrees (113). This should attract the attention of vaccine designers.

#### 3.2.2 ACP vaccine

ACP vaccine comprises Ag85B, CFP21, and PPE18 proteins. Recent studies have demonstrated the safety of this vaccine in animal models. The amount and titer of specific antibodies in mice were increased significantly after immunization. The vaccine increased the number of CD4^+^ T cells and secretion of the Th1-type cytokines IL-2 and IFN-γ significantly. The bacterial load in mouse lungs was decreased, and the pathological damage tended to be alleviated (114). Those effects may have been due to the absence of appropriate adjuvants in the vaccine or the route and frequency of vaccination needed to be improved to improve immunogenicity.

#### 3.2.3 INP-0288-1196-0125

The INP-0288-1196-0125 recombinant vector vaccine was constructed by a “mosaic” ice nucleation protein (INP) on the surface of *Escherichia coli*, and then Rv0288, Rv1196 (PPE18), and Rv0125 were expressed on INP. Without eliciting adverse reactions, this vaccine induced strong humoral immunity and CD4^+^ T-cell immune responses in mice. High levels of specific IgG could be detected after the first immunization. With an increase in immunization times, the number of CD4^+^ T cells and CD8^+^ T cells also increased, and the IL-4 level increased most significantly (115). ACP and INP vector vaccines show good tolerance and immunogenicity in mice and can induce a high level of immune response *in vivo*. In the next step, different suitable animal models can be found to test the characteristics of the vaccines multiple times to shorten the time required for the vaccines to enter clinical trials. A study on PPE18 suggests that analyses of the variability of protein–subunit sequences in vaccine candidates should not be neglected to improve the potential of vaccine-induced protective immune responses.

### 3.3 PPE42-related vaccines

#### 3.3.1 ID93:GLA-SE

PPE42 is also a valuable candidate protein for vaccines. PPE42 is involved in the construction of various vaccines, of which the most advanced is ID93:GLA-SE. ID93 is composed of Rv1813, Rv2608 (PPE42), Rv3619, and Rv3620. GLA-SE is an adjuvant containing the TLR4 agonist glucopyranosyl lipid A (GLA) and emulsifying stabilizer SE (116). This vaccine can enhance BCG immune outcomes and enhance drug efficacy as first-line treatment in patients with *Mtb* infection (117). ID93 showed immunogenicity in a variety of animal models (mice, guinea pigs, rhesus monkeys) and induced a multifunctional CD4^+^ Th1 cell response characterized by IFN-γ, TNF-α, and IL-2, which reduced the number of bacteria in the lungs of drug-resistant *Mtb* strains that attacked the lungs of the animals, and effectively lowered the mortality rate of tuberculosis in experimental animals (118). In phase I trials, the vaccine induced high-titer specific IgG (predominantly lgG1 and lgG3 (119), as demonstrated in dose-escalation trials) and Th1-cell responses *in vivo* in healthy BCG-naive adults, and vaccine-related serious adverse events were not observed (120). In a phase IIa trial in Cape Town (South Africa) involving adults with a history of TB, ID93 was shown to be safe and efficacious in improving treatment outcomes and preventing TB recurrence (121). ID93 has also been shown to reduce the bacterial load in the lungs of *Mtb*-infected mice effectively (122). Sixteen weeks after challenge with a hypervirulent Beijing strain of *Mtb* in BCG-immunized mice, ID93 could induce robust and sustained CD4^+^ T-cell responses and provide long-term, high-level protection against *Mtb* infection (123). Recently, researchers administered a dry-powder vaccine via intranasal and intralung routes in *Mtb*-infected mice. They found that ID93:GLA-SE could control inflammation progression, and detected a significant increase in the number of T cells and related cytokines. The immunogenicity and protective effect of ID93:GLA-SE were similar to those after intramuscular injection (124).

#### 3.3.2 Ag85B-EAST-6-PPE42 (rBCG)

The more studied vaccine target proteins Ag85B and ESAT-6 can also be fused with PPE42 to form a new recombinant BCG vaccine (rBCG) called Ag85b-ESAT-6-PPE42. rBCG can induce a stronger Th1-type cellular immune response and antigen-specific humoral immune response in an animal model compared with BCG. This vaccine has been shown to promote the proliferation of CD4^+^ T cells/CD8^+^ T cells, increase the level of IL-2/TNF-α significantly, and inhibit the secretion of the Th2-type cytokine IL-10. Meanwhile, an increase in IgG titer and IgG2b/IgG1 ratio has been observed (125).

#### 3.3.3 Tri-Fu64

Some researchers have recombined PPE42 with the *Mtb*-related virulence factor Rv1793 and latent antigen Rv2628 into a Tri-Fu64 vaccine. The latter can reduce the number of bacteria in the lungs of aerosol *Mtb*-infected mice and induce a certain degree of protective immunity. However, although Tri-Fu64 combined with the adjuvants MPL or dimethyl dioctadecyl ammonium bromide (DDA) can also improve the anti-infection ability of mice, different degrees of body weight loss were found in mice (126). This observation is a reminder that, in evaluating a vaccine, the focus should be not only on the immunogenicity of the vaccine and that attention should be paid to the possible rare types of adverse reactions. In addition, how to use the vaccine with the appropriate adjuvant should also be considered.

### 3.4 PPE44-related vaccines

PPE44 has multiple immunodominant T-cell epitopes and is involved in T-cell activation. The artificially prepared recombinant PPE44 protein (rPPE44) is a protective antigen that can stimulate cellular solid and humoral immunity in mice and induce similar protection to that seen with BCG (127). Among the vaccines developed using PPE44 as a candidate protein, research has focused on HPE. HPE comprises Rv2031c with the properties of heat shock protein X (HspX), the ESAT-6 family member Esx-V, and PPE44. This vaccine has been shown to enhance the primary immune response induced by BCG, and in addition to the increase in the IFN-γ level, the secretion of IL-12 and TGF-β in a suspension of spleen cells increased significantly. The application of the pDNA-HspX-PPE44-EsxV vaccine was safe, and no intolerance was observed in the injected mice throughout the experiment. Those results indicate that HPE can activate Th1 cells effectively and has advantages in maintaining the cellular immunity of T cells for a long time (128). To enhance the immunogenicity of HPE, HPE was combined with the TLR7/8 agonist resiquimod as an adjuvant and conjugated to chitosan nanoparticles to form the HPERC vaccine. Resiquimod is a potent, safe, and simple vaccine adjuvant. It can induce a powerful immune response, exhibiting effective antitumor effects in a murine melanoma model. It is considered to have great potential not only in tumor immunotherapy but also in infectious diseases caused by intracellular pathogens (129). This vaccine was injected (s.c.) into mice immunized with BCG at 1 and 2 weeks. High levels of specific cytokines, such as IFN-γ, IL-4, and IL-17, and significant humoral immune responses were observed, among which IgG2a content and titer increased the most (130). Although the level of the inhibitory factor IL-4 was also increased, this may be a mechanism to regulate the immune response to limit excessive inflammatory damage. A combination of HPE and the lipid adjuvants DDA and trehalose 6,6-dibehenate (TDB) could also enhance the protective efficacy of BCG and induce strong anti-*Mtb* cellular immune responses (131, 132). There is no doubt about the safety of DDA and TDB. In the tuberculosis vaccine currently in phase II clinical trials, H1 is combined with CAF01 adjuvant composed of DDA and TDB, and phase I clinical trials have confirmed that the vaccine containing DDA and TDB has excellent safety (133) and can improve the humoral immune response in AIDS patients (134). In conclusion, the HPE vaccine with PPE44 as a component showed good safety and immunogenicity in mice, could be used as a BCG booster vaccine, and could be improved further for clinical trials.

### 3.5 PPE57-related vaccines

PPE57 (Rv3425) is also a PE/PPE family member with strong immunogenicity and specificity. Immunization of mice with PPE57 protein was shown to increase IFN-γ production significantly and induce a strong IgG1 antibody response, leading to Th1- and Th2-type responses (135). Studies have shown that the degree of specific lgG response induced by artificially recombinant rPPE57 was higher than that induced by ESAT-6 and identical to that caused by CFP-10. Hence, PPE57 could be a vaccine candidate (136).

#### 3.5.1 Tetrafu56

The fusion peptide tetrafu56 was constructed by combining Rv3615c, TB10.4, PPE57, and HspX (a protein with high expression in the latent phase of *Mtb*). These four antigens contain a small number of specific T-cell epitopes. Their construction into a fusion protein can enable interaction with T cells and amplify the immune effect. This vaccine has been shown to induce high levels of protective IFN-γ (average = 397 pg/mL) from the PBMCs of patients with active pulmonary TB, but PBMCs from healthy adults were not sensitive to the vaccine and induced a low level of IFN-γ (average = 26.0 pg/mL) (137). Tetrafu56 (which incorporates PPE57) is a multiphase vaccine against TB composed of antigens from the active replication and resting stages of *Mtb*. Although it does not have a preventive effect, tetrafu56 has been shown *in vitro* to be more effective in interacting with PBMCs exposed to *Mtb* antigens, achieving adequate protection in patients with TB in a genetically heterogeneous population. In the next step, appropriate animal models can be selected to verify the immunological characteristics of the vaccine *in vivo*.

#### 3.5.2 A3 vaccine

The enhancement effect of PPE57 (Rv3425) is greater than that of Ag85B. The former can help maintain the body weight of mice after *Mtb* infection and has a longer-lasting protective effect on intravenously challenged mice (138). This fusion protein (whether carrying a virus or DNA vector) can increase the defensive efficacy of BCG (139). The Ag85B-Rv3425 (A3-lentivirus, abbreviated as “A3-len”) vaccine is constructed by the lentivirus vector. A single dose of A3-len has been shown to stimulate the proliferation of CD4^+^ T cells and reduce the number of CD8^+^ T cells, as well as induce high levels of IFN-γ, IL-2, TNF-α, and A3-specific IgG. The CFU count and pathological examination showed that the vaccine could reduce the bacterial population in the lungs and spleen by inhibiting the growth and reproduction of *Mtb in vivo*. A3-len can reduce the severity of lung tissue lesions, increase the body weight of mice with active TB gradually, fight against the injury wrought by acute *Mtb* infection, and provide immune protection for mice (140, 141). Based on the A3 platform, Su et al. added the latent period protein Rv2029c to it to form the A39 vaccine. Rv2029c and PPE57 are the same as the crucial components of this vaccine. The former can increase the antigen presentation ability of CD4^+^ T cells, stimulate macrophages to activate T cells so that they secrete large amounts of IFN-γ and IL-2 to maintain immune memory, and help T cells to polarize to Th1 cells (142). The most important feature of A39 is that it can control bacterial replication in organs, and the inhibitory effect of A39 on bacterial load reactivation is higher than that of drugs used for TB therapy. Several studies have shown the advantages of A3 in mice. A3 could be used as a “platform” to screen and add safer and highly immunogenic antigen-modified vaccines and improve efficacy.

### 3.6 PPE68-related vaccines

PPE68 (Rv3873) is one of the proteins encoded by the region of differences (RD) 1 region of the H37Rv strain. As an immunodominant antigen, it is involved in antigen diversity and immune escape of *Mtb* but is unrelated to the virulence of the RD1 region.

#### 3.6.1 PPE68-rBCG

PPE68 expression in BCG without an RD1-related protein constitutes a safe PPE68-rBCG which can induce a higher Th1 response than that elicited by BCG alone. The levels of IFN-γ, IL-12, and IgG2a and the splenic CD4^+^ T-cell count were increased significantly as measured by enzyme-linked immunosorbent assays, and the ratio of CD4^+^ T cells/CD8^+^ T cells decreased (143). Insertion of the fusion proteins PPE68, CFP-10, and ESAT-6 into the plasmid vector was shown to stimulate a large amount of IFN-γ release in the blood of patients with active pulmonary TB *in vitro*. Murine experiments also showed that the fusion protein could increase the titers of IFN-γ and lgG and maintain a long duration of humoral immunity (144).

#### 3.6.2 rLmMtb9Ag

This recombinant vaccine is composed of *Listeria monocytogenes* and nine antigens (including PPE68). The safety and immunogenicity of rLmMtb9Ag were evaluated in mice and guinea pigs without primary vaccination using BCG. This multi-antigen vaccine induced the proliferation of antigen-specific CD4^+^ T cells and CD8^+^ T cells, reduced the CFU count of *Mtb* in the lungs and spleen, and produced protective immunity in guinea pigs infected with an aerosol of the hypervirulent Erdman strain of *Mtb* (145). The high immunogenicity of PPE68 may be due to its P9 peptide composed of amino acids 121–145, which is highly conserved in pathogenic mycobacteria (146). P9 can be purified by genetic engineering and used as a candidate subunit of a vaccine against TB.

### 3.7 PE-PGRS33-related vaccines

PE family proteins are rich in PGRS, so they are expressed constitutively only in pathogenic mycobacteria and are essential for the basic functions of bacteria. The most well-studied protein is PE-PGRS33 (Rv1818c), which is associated with long-term latent infection with *Mtb*. The PE segment is inserted into the cell wall and is necessary for *Mtb* to transport and localize proteins through the cell wall (147, 148). PGRS are partially located in the extracellular domain, in which other antigens can be inserted into the “sandwich domain” PG II without affecting the stability of their structure (149). Therefore, the immunogenicity of the protein can be increased by modification such as insertion. Bioinformatics analysis has shown that PE-PGRS33 contains 27 B-cell- and four T-cell-dominant epitopes, thereby significantly stimulating a highly effective humoral immune response. Delogu and colleagues first isolated PE and PPE fragments and cloned the PE sequence. Inoculation of rPE into mice could stimulate the proliferation of mouse T cells and secrete IFN-γ, and specific antibodies could be obtained when the intact PE-PGRS33 was inoculated (150). The PE-PGRS33 antibody was conjugated onto the surface of *Mtb*, which could bind to TLR2 to inhibit *Mtb* entry into macrophages and its proinflammatory activity, block the pathogenic pathway of TB, and promote activation of macrophages as well as the effective uptake and killing of bacteria (151). The PE segment and MPT64 could be combined to form rBCG. Due to the transport function of the PE segment, MPT64 could be delivered to the cell surface, providing higher protection efficiency than BCG, reducing the number of bacteria in the lungs and spleen, and stimulating the proliferation of CD4^+^ T cells and CD8^+^ T cells and the release of IFN-γ (152). Subsequent experiments demonstrated that IFN-γ and specific antibodies against Rv1818c were also observed in patients with LTBI and healthy adults immunized with BCG (44). Those results suggest that the PE fragment can induce protective cellular immunity and that the development of a vaccine formulation associated with an anti-PE-PGRS33 antibody may help suppress inflammation and prevent TB progression.

## 4 Discussion

In recent years, the increasing incidence of TB worldwide has incited the need to prevent the disease. The research and development of vaccines against TB have been at the forefront of this strategy. PE/PPE is a multifunctional protein family of *Mtb* with a wide range of members and complex sequences. PE/PPE proteins are involved in the interaction between pathogens and macrophages and play essential roles in immune recognition, immune escape, and pathogenicity of *Mtb*. The application of bioinformatics analysis has enabled the prediction and understanding of the biological structure of PE/PPE proteins as well as the crucial roles of PE/PPE in *Mtb* infection. Also, understanding the targets and processes of the interaction between PE/PPE proteins and immune cells will aid in the screening of antigens for the development of new vaccines against TB. PE/PPE-related vaccines are representative of subunit vaccines, and PE/PPE proteins are combined with adjuvants or other vectors, which have both stability and safety. It is designed to enhance the immune response after BCG vaccination and prolong the duration of protection. Due to the importance of PE/PPE family proteins in the pathogenesis of *Mtb*, more and more tuberculosis vaccines are designed to include PE/PPE family proteins to further improve the immune effect. Among the PE/PPE protein vaccines, M72 and ID93 have made rapid progress and are about to enter phase III clinical trials. Both vaccines are well tolerated in the subject population and can effectively control the progression of inflammation and the recurrence of tuberculosis. Other PE/PPE vaccines have also shown outstanding safety and immunogenicity in animal trials and have made remarkable achievements, with great hope to enter the clinical trial stage. ‘Taking BCG as the “gold standard”, the long-term safety and immunoprotective development of other vaccines against TB should be as good as that of BCG’ is a recognized principle of the International Organization for the Prevention of Tuberculosis. The difficulty in developing a new vaccine against TB is that the pathogenesis of TB and the immune response to *Mtb* infection are incompletely understood. Further understanding of the mechanism of action of *Mtb* has profound importance for vaccine development. In addition, an appropriate adjuvant can increase the immunogenicity and optimize the targeted delivery of antigen based on reducing the antigen dose. Hence, choosing an appropriate adjuvant for use with the vaccine is also crucial.

The increasing incidence of TB has brought heavy political and economic burdens to developing countries. The international community and public welfare organizations should increase investment in the research and development of vaccines. In 2018, the WHO proposed a vaccine to prevent TB in adults that should achieve >50% protection (153). This requirement also increases the standard and difficulty of vaccine development. However, with the rapid development in immunology and molecular biology, we believe that, through in-depth research and optimization of vaccines against TB, eliminating TB by 2050 is achievable. In conclusion, we believe that the PE/PPE family will remain a highly active and promising area of research and that their potential as TB vaccine targets will continue to be exploited, with more exciting properties to be explored.

## Author contributions

FG: Data curation, Formal Analysis, Investigation, Project administration, Writing – original draft. JW: Investigation, Formal Analysis, Methodology, Writing – original draft. YS: Investigation, Writing – original draft, Methodology. BL: Writing – review & editing. ZQ: Methodology, Supervision, Writing – review & editing. XW: Writing – review & editing, Supervision, Validation. HW: Funding acquisition, Resources, Supervision, Writing – review & editing. TX: Funding acquisition, Investigation, Resources, Supervision, Writing – review & editing.
